# Splenic Pedicle Volvulus in a Wandering Spleen: A Rare Pediatric Emergency

**DOI:** 10.7759/cureus.90043

**Published:** 2025-08-13

**Authors:** Nusrath M P, Mina Raad Al Mnaseer, Sarah Siddiqui, Asiya Mubeen, Yusra Jamil

**Affiliations:** 1 Pediatric Emergency Medicine, Al Jalila Children's Speciality Hospital, Dubai, ARE; 2 Medicine, Dubai Medical University, Dubai, ARE

**Keywords:** splenectomy, splenic infarction, torsion, volvulus, wandering spleen

## Abstract

Wandering spleen is a rare clinical condition caused by laxity or absence of splenic ligaments, which allows for abnormal splenic mobility and potential torsion. Splenic torsion, the most serious complication, accounts for many symptomatic cases. We describe the case of a 13-year-old girl who had vomiting, fever, and severe abdominal pain. On examination, she was febrile and tachycardic, while other parameters were within normal limits. Abdominal examination revealed tenderness in the left upper quadrant. Imaging showed a wandering spleen and torsion with impaired blood flow and evidence of infarction. An emergency splenectomy was performed due to the total loss of splenic perfusion. This case underscores the importance of early imaging and prompt surgical intervention to prevent irreversible ischemic injury and associated morbidity.

## Introduction

During an autopsy in 1667, Dutch surgeon Van Horne was the first to describe the wandering spleen [1]. The wandering spleen can also be referred to as aberrant, floating, ptotic, displaced, or prolapsed. The spleen exhibits excessive mobility, showing an elongated pedicle and displacement from its normal position in the left upper quadrant. The possible causes of this pathology are embryologically absent or malformation of one or more suspensory ligaments, and the laxity of the abdominal musculature weakened by pregnancy hormones, or failure of dorsal peritoneal fusion [2]. Wandering spleen is a rare clinical condition, allowing the spleen to move from its normal anatomical position to other areas of the abdomen or pelvis [3]. Although it may remain asymptomatic, torsion of the splenic pedicle may occur, leading to infarction and splenic rupture. Clinical presentation is unpredictable and often nonspecific, including intermittent or acute abdominal pain, nausea, vomiting, and occasionally a palpable abdominal mass [4]. Splenic torsion is a complication in 64% of children with wandering spleen, although its clinical presentation varies [3]. The diagnosis can be difficult and often delayed because of its rarity and vague signs and symptoms. Management depends on splenic viability at the time of diagnosis. Early recognition and surgical intervention are critical to avoid irreversible ischemic damage and reduce postoperative morbidity. This report presents a case of acute splenic torsion in a 13-year-old girl with a previously identified ectopic spleen, emphasizing the importance of early recognition and timely surgical management of splenic torsion.

## Case presentation

 A 13-year-old, previously healthy teenage girl presented to the emergency department (ED) with a six-day history of abdominal pain, which was generalized but predominantly localized to the epigastric region. The pain was described as aching, constant in nature, and rated 5/10 in severity. It was associated with a four-day history of fever, constipation, nausea, and non-projectile, non-bilious, non-bloody vomiting. She had no urinary, respiratory, or gynecological symptoms. She visited the ED two days ago with similar complaints and was discharged after symptomatic improvement with analgesics. Her past medical history was unremarkable except for an episode of cystitis three years ago and an incidental finding of a wandering spleen on abdominal imaging. The patient failed to show up despite being advised to follow up and to decide on a further management plan. There was no relevant family history. On examination, she was alert but appeared tired. Her vital signs revealed a fever of 38.7 °C and tachycardia, while other parameters were within normal limits. Heartrate-122/mt, regular, Respiration-24/mt, regular, Blood pressure-113/68mmHg, oxygen saturation-100% in room air. Abdominal examination revealed tenderness, particularly in the left upper quadrant, but the abdomen was soft without guarding or rigidity. We considered the possibility of sepsis, pancreatitis, acute gastritis, and splenic torsion because of earlier ultrasound findings and proceeded with investigations.

Initial laboratory investigations revealed leukocytosis, neutrophilia, and elevated C-reactive protein (CRP) and procalcitonin, suggestive of systemic inflammation, potentially indicating torsion/infarction. Mild anemia and platelets towards the lower limit may indicate impaired spleen function. Both urine and blood cultures were normal. Liver function tests, renal profile, and serum amylase were within normal limits (Table 1).

**Table 1:** Blood investigations WBC: White Blood Cells; RBC: Red Blood Cells; MCV: Mean Corpuscular Volume; MCH: Mean Corpuscular Hemoglobin; MCHC: Mean Corpuscular Hemoglobin Concentration; RDW: Red Cell Distribution Width; MPV: Mean Platelet Volume; SGOT (AST): Aspartate aminotransferase; SGPT (ALT): Alanine aminotransferase; PCT: Procalcitonin

Component	Reference Range	Result
WBC Count	5.0 - 13.0 10^3/uL	11.3
RBC Count	4.00 - 5.20 10^6/uL	3.98
Hemoglobin	11.5 - 15.5 g/dL	10.8 Low
Hematocrit	35.0 - 45.0 %	33.4
MCV	77.0 - 95.0 fL	83.9
MCH	25.0 - 29.0 pg	27.1
MCHC	31.5 - 34.5 g/dL	32.3
RDW	11.5 - 14.0 %	14.2
Platelets Count	170 - 450 10^3/uL	196 Low
MPV	7.4 - 10.4 fL	11
Neutrophil Absolute	2.0 - 8.0 10^3/uL	8.02
Lymphocytes Absolute	1.00 - 5.00 10^3/uL	2.24
Monocytes Absolute	0.20 - 1.00 10^3/uL	0.76
Eosinophils Absolute	0.10 - 1.00 10^3/uL	0.18
Basophils Absolute	0.00 - 0.10 10^3/uL	0.05
Neutrophil %		71.3
Lymphocyte %		19.9
Monocyte %		6.8
Eosinophil %		1.6
Basophil %		0.4
Sodium	136 – 145 mmol/L	139
Potassium	3.5 - 5.1 mmol/L	3.6
Chloride	97 - 107 mmol/L	109
Bicarbonate (HCO3)	17 - 27 mmol/L	19
Creatinine	0.52 - 0.69 mg/dL	0.66
Urea	19.26 - 47.294 mg/dL	16
Calcium	8.8 - 10.8 mg/dL	8.6
Glucose, Random	73 - 112 mg/dL	96
SGOT (AST)	0 - 51 U/L	18
SGPT (ALT)	0 - 39 U/L	8
Alkaline Phosphatase	153 - 367 U/L	89
Total Protein	6.4 - 7.7 g/dL	6.5
Bilirubin, Total	0 - 1.2 mg/dL	0.52
Albumin	3.8 - 5.4 g/dL	4
C-Reactive Protein	0 - 5 mg/L	242.3 High
PCT	< 0.5 ng/mL	0.16 High

**Figure 1:**
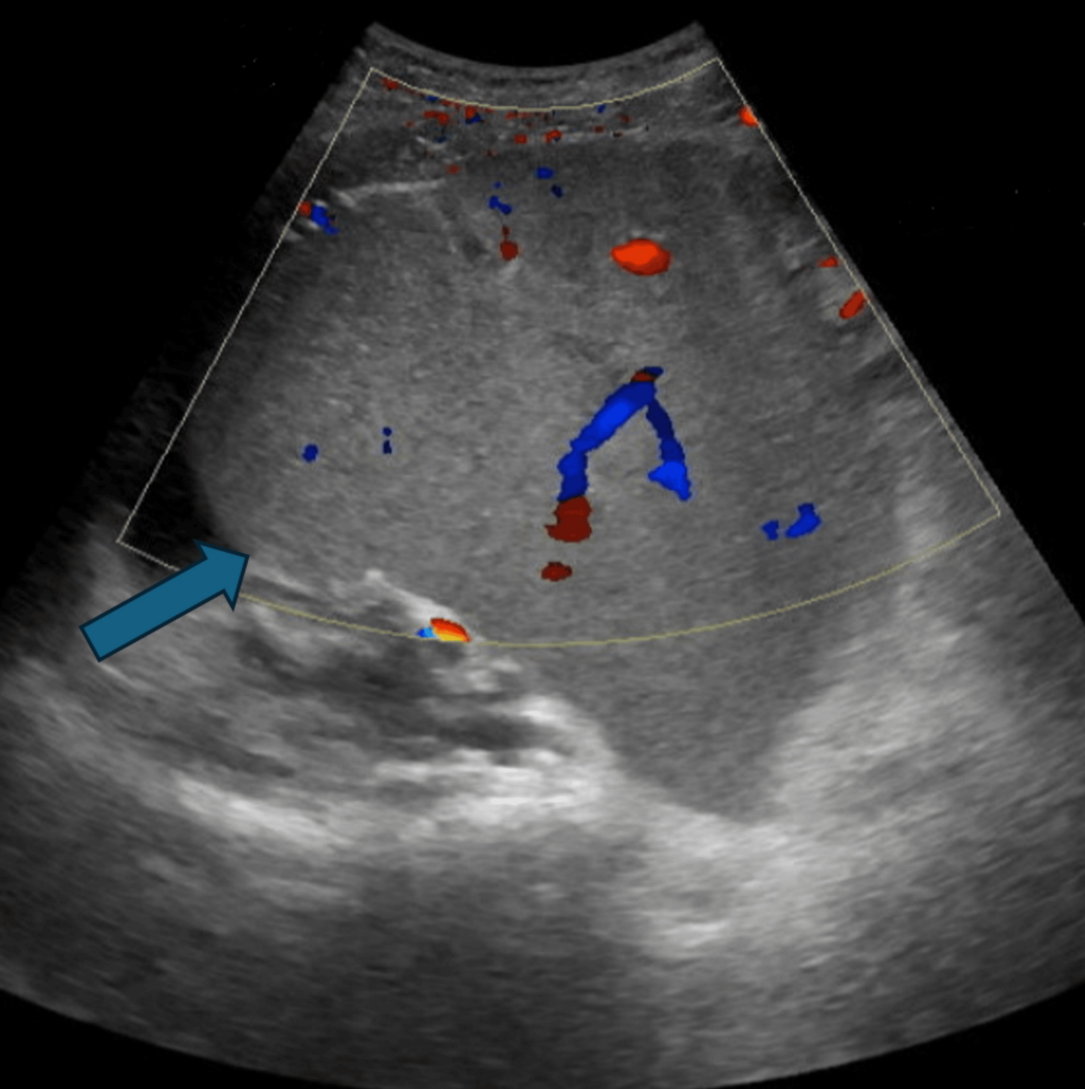
Ultrasound showed enlarged spleen located in the right lower abdomen and pelvis in the subumbilical region, with color doppler flow seen in the splenic hilum and parenchyma Blue arrow: spleen

**Figure 2:**
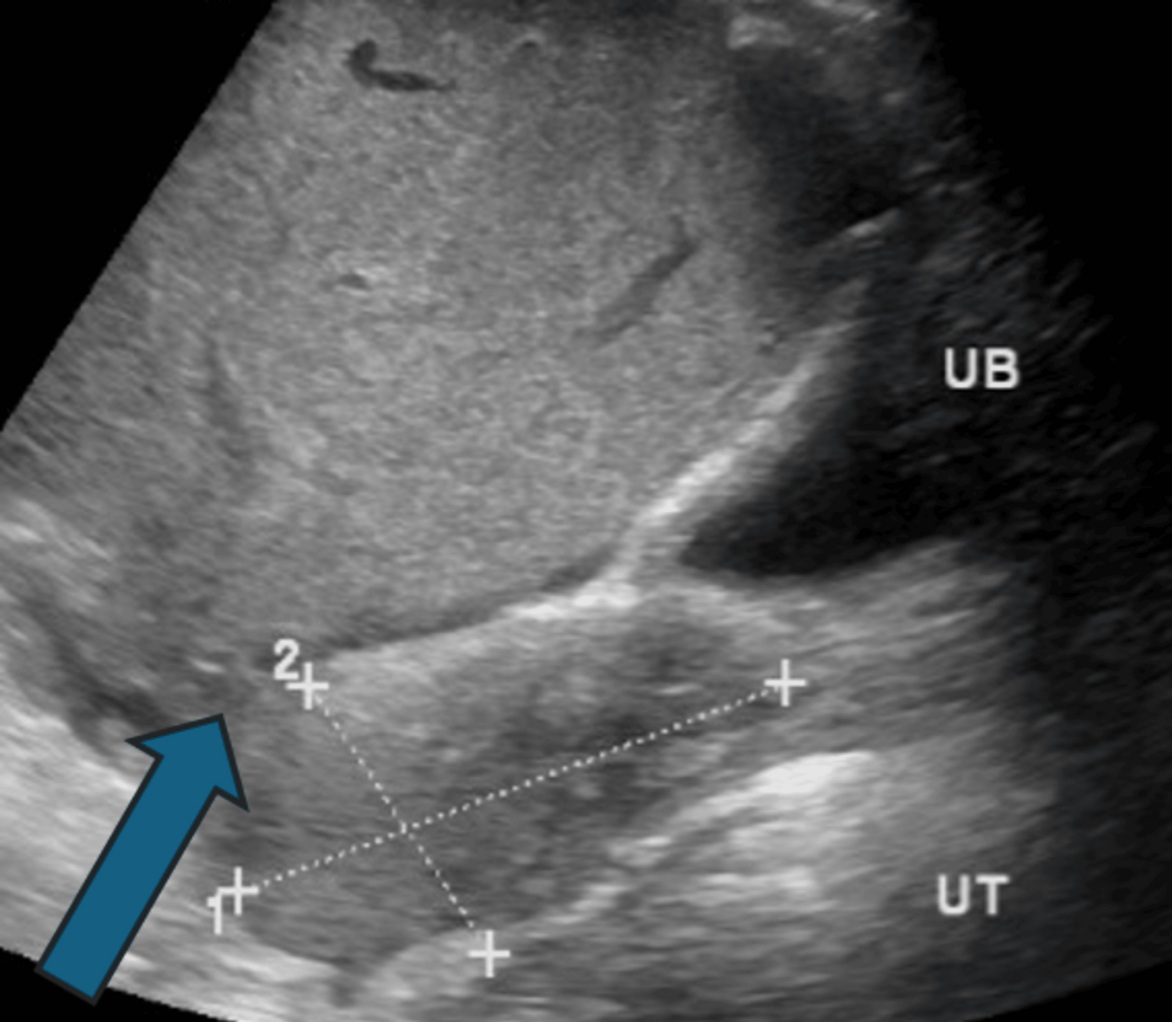
Ultrasound showing abnormal position of the spleen in the pelvis Blue arrow: spleen

Abdominal ultrasonography demonstrated an ectopic, enlarged spleen located in the right lower abdomen and pelvis in the subumbilical region, with color doppler flow seen in the splenic hilum and parenchyma. The splenic artery showed a low-volume, parvus tardus flow pattern. Splenic vein flow is seen. An echogenic lesion seen in the left side of the abdomen with a whirlpool sign indicative of torsion of the splenic vascular pedicle/splenic volvulus was also observed. Liver is normal, measures 14.8 cm, with a homogenous pattern and smooth, regular surface (Figures 1, 2).

**Figure 3:**
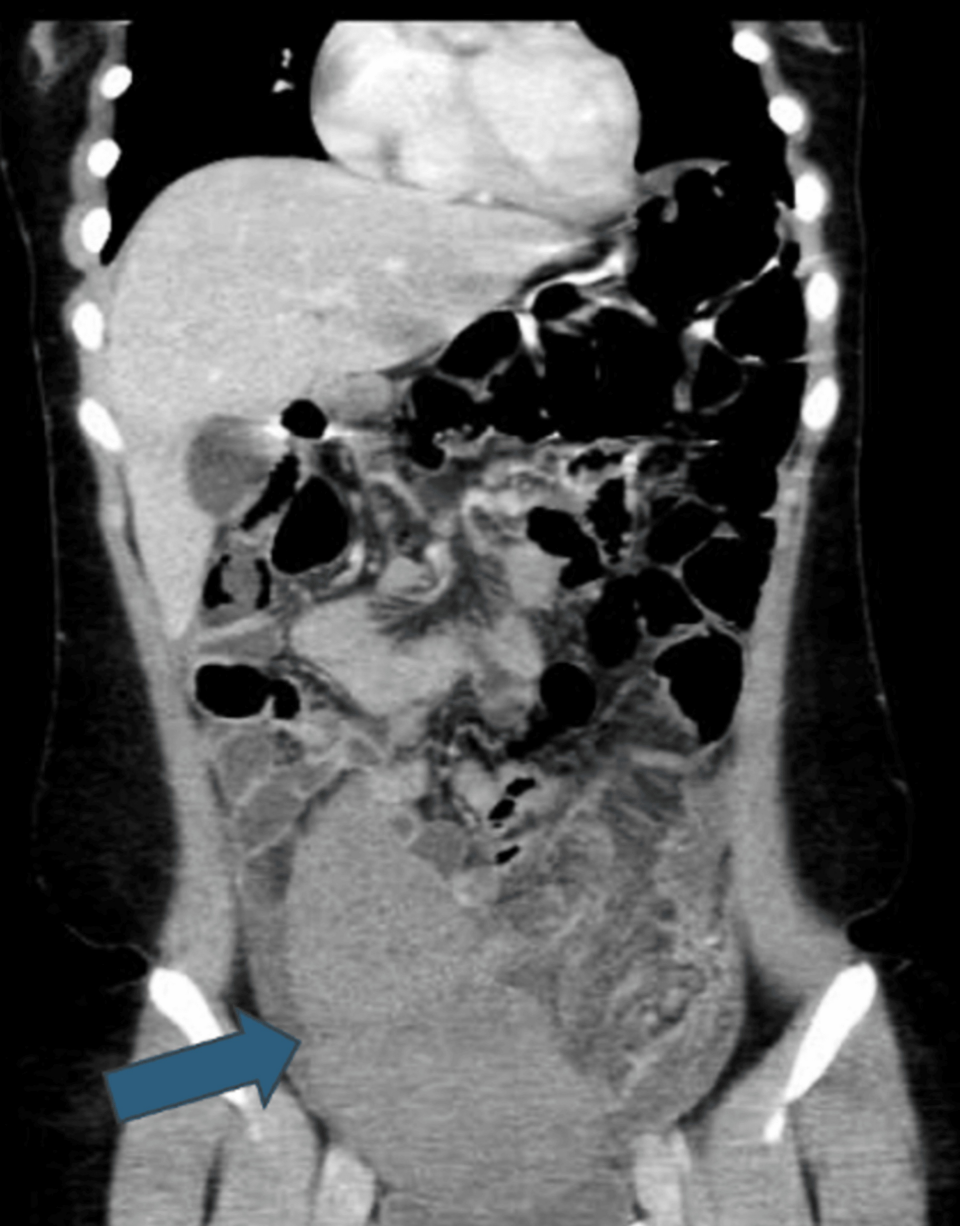
The coronal plane showing the abnormal location of the spleen in the right lower abdomen, with signs of torsion of the spleen Blue arrow: spleen

**Figure 4:**
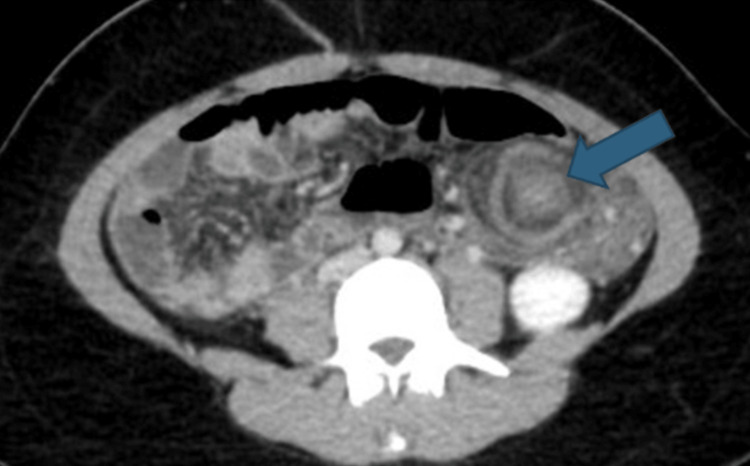
The axial plane of contrast-enhanced computed tomography images showing an enlarged spleen with a low position Blue arrow reveals a twisting of the splenic vessels “whirlpool sign”

**Figure 5:**
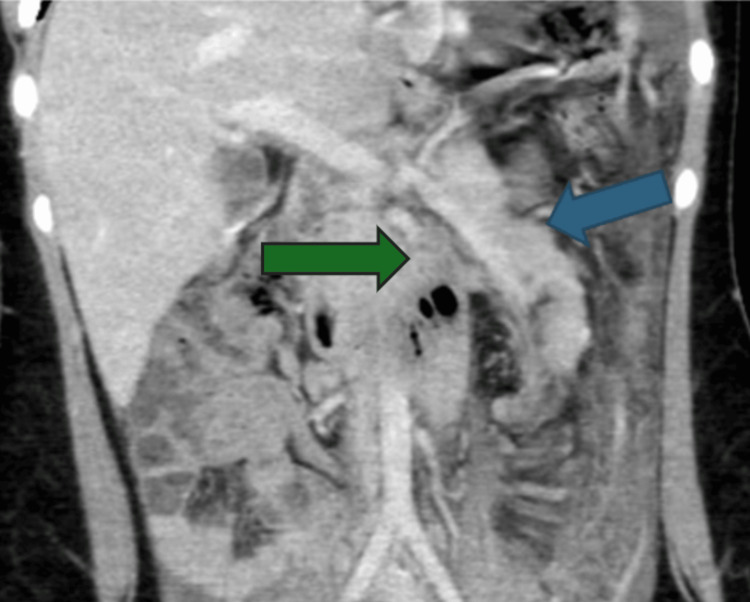
Contrast CT showing the ectopic spleen and the twisted splenic vascular pedicle (whirl sign) Green arrow: ectopic spleen; Blue arrow: splenic vascular pedicle

A contrast-enhanced CT scan of the abdomen confirmed the presence of a wandering spleen measuring 16.6 cm in the pelvis and right lower abdomen, with evidence of vascular pedicle twisting suggestive of volvulus. There is twisting of the vascular pedicle along with the distal body and tail of the pancreas, seen on the left side of the abdomen. Mild homogenous enhancement observed in the spleen. A non-enhancing linear area on the right lateral aspect of the spleen was suggestive of infarction, and there was associated free fluid in the left subdiaphragmatic and right lower abdominal regions (Figures 3, 4, 5).

**Figure 6:**
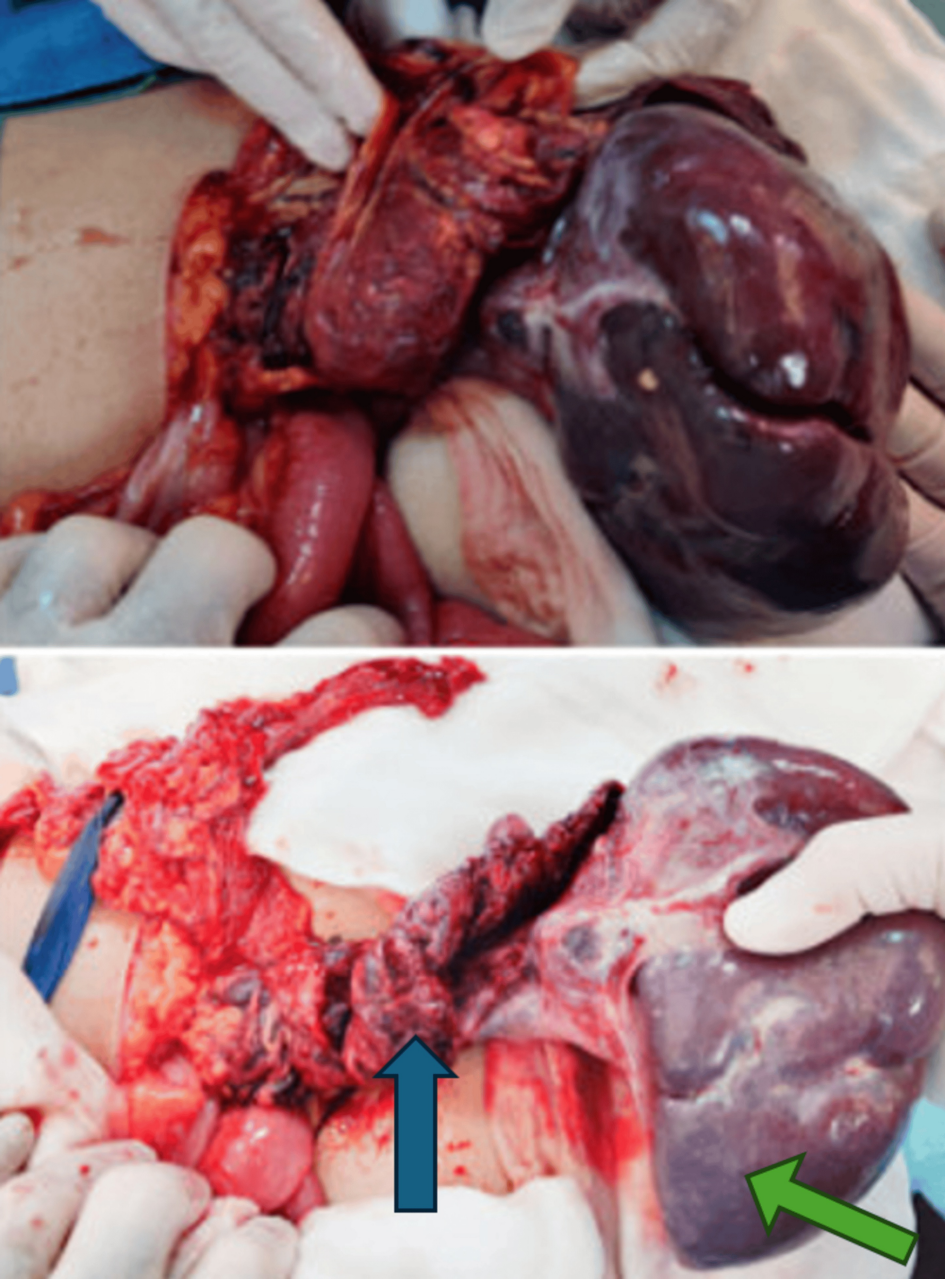
Surgical specimens showed the torsion of the splenic pedicle (blue arrow). Areas of infarction and necrosis (green arrow)

Given the imaging findings, an urgent splenectomy was recommended. However, the surgery was postponed for 24 hours due to parental hesitation. The following day, under general anesthesia, a 10 mm umbilical port was inserted for diagnostic laparoscopy, which revealed a wandering spleen located in the pelvic cavity, appearing enlarged, discolored, and with necrotic patches. The spleen was found to have multiple twists of its pedicle and involvement of the omentum. Given these findings, the procedure was converted to an open laparotomy via a left transverse upper abdominal incision. On detorsion, the splenic vessels were thrombosed with no evidence of reperfusion, confirming non-viability. A total splenectomy was performed (Figure 6).

The patient tolerated the procedure well. She was monitored postoperatively, and recovery was uneventful apart from mild, intermittent pain at the surgical site. She was discharged on the third postoperative day in a stable condition on penicillin prophylaxis with instructions for follow-up care. In accordance with current guidelines, she received pneumococcal and meningococcal vaccinations 14 days post-splenectomy. Histopathology reported as the overall histology composed of expanded red pulp (occupies ~ 75% of splenic volume) and white pulp separated by the marginal zone. Red pulp is expanded and shows thin-walled venous sinusoids lined by littoral-like cells, and sinuses separated by splenic cords. Focal hemorrhage as well as numerous red blood cells, prominent vascular proliferation with round and irregularly shaped vascular spaces, and anastomosis with focal infarction/ thrombi. The white pulp forms sheaths of lymphoid cells around arteries (periarteriolar lymphatic sheath). Negative for carcinoma and lymphoproliferative neoplasm.

## Discussion

The spleen is an intraperitoneal organ that is connected to the stomach and the back of the abdomen by the gastrosplenic ligament, which contains the splenic artery, vein, and associated lymphatic vessels, as well as the splenorenal ligament [1]. The splenocolic and splenophrenic ligaments support it inferiorly, and the splenopancreatic ligament connects it to the pancreatic tail. These peritoneal ligaments can hold the spleen in the left hypochondrium. In wandering spleen, the spleen moves from the left upper quadrant to ectopic places, most frequently the hypogastrium. This rare disorder can be acquired or congenital [5]. Elongated spleen’s vascular pedicle, which functions as an axon and, prone to torsion, causes hypoperfusion of the splenic parenchyma, congestion, thrombosis, and ultimately infarction and necrosis of the spleen [6]. The overall incidence is less than 0.2% with two incidence peaks between three months and 10 years and between 20 and 40 years [3,7,8]. Wandering spleen is a rare cause of acute abdomen in children, accounting for less than 0.2 % of splenectomies [9]. Patients may be asymptomatic, present with a movable lump in the abdomen, or exhibit acute, chronic, or intermittent symptoms because of torsion of the wandering spleen. Chronic presentations may involve vague abdominal discomfort, and acute presentations typically mimic other surgical emergencies, complicating early diagnosis. Gastric volvulus, variceal hemorrhage, and acute pancreatitis, which is caused by an illness of the pancreatic tail limited to the lienorenal ligament, are other uncommon conditions linked to a wandering spleen [10,11]. Our case presented with epigastric pain, but the amylase was normal. When splenic torsion occurs, patients may have leukocytosis and elevated C-reactive protein [12]. In our case, elevated C-reactive protein and leukocytosis were present.

Imaging tests serve as the foundation for diagnosis because laboratory tests and clinical presentations are nonspecific. Imaging modalities are crucial because they can be challenging to make an early clinical diagnosis [13]. The clinical importance of this case lies in the delayed presentation and the diagnostic reliance on imaging modalities, particularly the identification of the “whirlpool sign”, a hallmark of splenic pedicle torsion [14]. Although often diagnosed incidentally or intraoperatively, improved imaging access has enabled earlier noninvasive recognition. In our case, ultrasonography also revealed a whirlpool sign. Ultrasound with Doppler remains a frontline tool, though contrast-enhanced CT provides greater anatomical detail, especially when assessing for infarction, vascular compromise, or displacement of adjacent structures to facilitate surgical planning [3,14]. As wandering spleen can mimic other pathologies, a CT scan is still the preferred test to rule out other causes of acute abdomen, as well as to confirm the diagnosis. In this case, the CT revealed twisting of the splenic pedicle with associated rotation of the distal pancreas, a finding that, while uncommon, underscores the extent of anatomical disruption that can accompany wandering spleen.

Management strategies hinge on splenic viability. Laparoscopic splenopexy is preferred to preserve immunologic function in pediatric patients; however, infarction or nonviability necessitates splenectomy [15]. In this instance, thrombosis and necrosis precluded preservation. Timely surgical intervention, even after a brief delay, prevented further complications such as hemorrhage or abscess formation. Laparoscopic splenectomy is currently advised and is frequently used as it has reduced postoperative pain and hospital stay, improved cosmetic outcomes, and lower overall morbidity rate [15]. In our case, we decided to have an open splenectomy due to extensive necrosis of the spleen parenchyma. All patients should receive pneumococcal, meningococcal, and Haemophilus influenza vaccinations following splenectomy [16]. Fourteen days following the splenectomy, our patient was vaccinated. In non-immunocompromised populations, US and Australian guidelines recommend antibiotic prophylaxis for one to three years after splenectomy or until five years of age; for those who are immunocompromised or with an episode of sepsis, lifelong prophylaxis is recommended [17]. The risks of infection following a splenectomy should be explained to patients and their families. Early detection of infection-related symptoms (fever, chills, rigors, vomiting, diarrhea) and prompt medical attention are essential [17].

This case reinforces the need for heightened clinical suspicion in pediatric patients with unexplained abdominal pain and systemic inflammation. Early imaging, multidisciplinary decision making, and pre-/postoperative planning, including immune prophylaxis,s are necessary for optimal results.

## Conclusions

Wandering spleen, though rare, should be considered in pediatric patients with nonspecific abdominal pain and systemic inflammatory signs. Its variable presentation and potential for life-threatening complications, including torsion and infarction, necessitate a high index of suspicion and early imaging-particularly ultrasound and CT-for timely diagnosis. Prompt surgical intervention is critical to prevent irreversible vascular compromise; splenoplexy is preferred in viable spleens, while splenectomy remains the treatment of choice in cases with infarction. Postoperative care, including vaccination and patient education, is essential for long-term management. Greater awareness among clinicians can facilitate early recognition and improve outcomes in this uncommon but serious condition.
